# Nighttime safety of daridorexant: Evaluation of responsiveness to an external noise stimulus, postural stability, walking, and cognitive function

**DOI:** 10.1177/02698811241293997

**Published:** 2024-12-06

**Authors:** Massimo Magliocca, Ingrid Koopmans, Cedric Vaillant, Vincent Lemoine, Rob Zuiker, Jasper Dingemanse, Clemens Muehlan

**Affiliations:** 1 Centre for Human Drug Research, Leiden, The Netherlands; 2 Leiden University Medical Centre, Leiden, The Netherlands; 3 Department of Clinical Pharmacology, Idorsia Pharmaceuticals Ltd, Allschwil, Switzerland; 4 Global Life Cycle Management, Idorsia Pharmaceuticals Ltd, Allschwil, Switzerland

**Keywords:** Orexin, nighttime safety, postural stability, awakening threshold, cognitive function

## Abstract

**Background:**

Daridorexant is a dual orexin receptor antagonist approved for the treatment of chronic insomnia disorder.

**Aims:**

Investigate the auditory awakening threshold (AAT), postural stability, and cognitive function during the night following evening administration of daridorexant 25 and 50 mg.

**Methods:**

Double-blind, placebo-controlled, randomized, 3-way (placebo, 25, 50 mg) crossover study in 36 healthy male and female nonelderly adult and elderly subjects (1:1 sex/age ratio). Four hours after bedtime administration, the AAT was determined, followed by investigation of the main pharmacodynamic endpoint nocturnal postural stability (body sway) as well as functional mobility using the Timed Up and Go (TUG) test, and cognitive function/memory using the Visual Verbal Learning Test (VVLT).

**Results:**

All 36 subjects completed the study. The average AAT was approximately 60 dB across treatments, i.e., there were no differences between daridorexant and placebo. Daridorexant marginally increased body sway by approximately 22%, while it had no clinically meaningful effect on the time to complete the TUG test (⩽1 s increase), and the VVLT (immediate and delayed number of correctly recalled words) showed minimal and clinically not meaningful differences of up to one word, all compared to placebo. Delayed word recognition was not different from placebo. The increase in body sway in the overall population was driven by nonelderly adults, as effects in elderly subjects were similar to placebo.

**Conclusions:**

Following bedtime administration, daridorexant maintained the ability to awaken to an external noise stimulus in the middle of the night, allowing subjects to function safely.

**ClinicalTrials.gov Identifier:**

NCT05702177.

## Introduction

Daridorexant is a selective dual orexin receptor antagonist (DORA) that equipotently inhibits both orexin-1 and orexin-2 receptors in wake-promoting brain regions, counteracting the hyperactive brain state in insomnia patients and eventually promoting sleep (Muehlan et al., 2020a, Mignot et al., 2022). Following market authorization in 2022 by the US Food and Drug Administration and subsequently by the European Medicines Agency, other European entities, and Canada, daridorexant is currently the only DORA available in Europe for the treatment of adult patients with insomnia (QUVIVIQ™ USPI, QUVIVIQ™ SmPC, Mignot et al., 2022). Standard-of-care therapies for insomnia include various nonpharmacological treatment options, such as maintaining good sleep hygiene measures, but also psychological and behavioral options, such as sleep restriction, stimulus control, and relaxation therapies, as well as cognitive behavioral therapy for insomnia (CBT-I) (Riemann et al., 2023; Schutte-Rodin et al., 2008). However, CBT-I may be difficult to access, or some patients may lack interest in CBT-I (Riemann et al., 2023). Therefore, pharmacological options are also needed.

Ideally, safe and effective pharmacological treatments of insomnia should facilitate initiation/maintenance of sleep while minimizing alterations of responsiveness, mobility tasks, or important cognitive functions. This ensures that patients can safely operate during the night if necessary (Drake et al., 2017). In this context, DORAs, which target wake-promoting neuropeptides that are primarily active during wakefulness and both promote and stabilize wakefulness (Muehlan et al., 2023; Roch et al., 2021), are of particular interest.

During early-stage development, daridorexant administered during the day to healthy individuals demonstrated expected pharmacodynamic (PD) effects. These included reduced peak velocity of saccadic eye movements, decreased adaptive tracking performance (a reactive eye–hand coordination task using a joystick), and increased static postural sway (Muehlan et al., 2018, 2019). Additionally, it was shown that pharmacokinetics (PK) and PD are timely related. For a given dose, the extent and duration of effects are explained by its PK profile, with PD effects returning to baseline 6–10 h after a 50 mg dose of daridorexant. This pharmacological PK/PD relationship was further investigated and confirmed through the observed clinical dose–response relationship for sleep parameters in patients with insomnia that were evaluated in Phase 2 and 3 clinical trials (Dauvilliers et al., 2020; Mignot et al., 2022; Zammit et al., 2020).

It is a well-known concern with central nervous system (CNS) depressant drugs that they may decrease the ability of insomnia patients to awaken to external stimuli such as smoke alarms or crying children, and impair balance, which is associated with an increased risk of falling (Drake et al., 2017; Fernie et al., 1982; Mets et al., 2010). The purpose of the present study was to investigate whether the PD properties of daridorexant, which translate into clinical benefit for insomnia, compromise the ability to awaken in response to an external noise stimulus and to safely function in the middle-of-the-night (MOTN) after bedtime administration of the two marketed doses, 25 and 50 mg.

## Methods

### Study population

The study was conducted in a total of 36 healthy male and female nonelderly adult (*N* = 18, aged 18–64 years) and elderly (*N* = 18, aged ⩾ 65 years) subjects with a balanced 1:1 sex/age ratio. The rationale of selecting healthy subjects was to ensure an accurate investigation of nighttime PD variables without the confounding factors of concomitant diseases or medication. Furthermore, this choice enabled comparison to previous findings obtained in healthy subjects when the PD of daridorexant were evaluated at different doses following daytime administration. Eligible subjects were required to have a usual bedtime between 21:30 and 00:30 and a regular sleep pattern with a sleep duration of at least 6 h. In addition, subjects were not eligible in case they had a history of narcolepsy, if they had travelled across ⩾3 time zones within 1 week prior to screening or planned this during the study, or if they had done shift work within 2 weeks prior to screening or planned to shift work during the study. Pregnant or lactating women were not allowed to participate in this study. Elderly subjects had to have a Mini-Mental State Examination score of ⩾25 to be considered eligible for this study. Following adequate explanation of the objectives, methods, and potential hazards associated with participation in this study, written informed consent was obtained from each subject prior to starting any study-related assessments or procedures.

### Study design and treatment administration

The study (ClinicalTrials.gov Identifier: NCT05702177) was conducted at the Centre for Human Drug Research (CHDR), Leiden, The Netherlands. The study design of this double-blind, placebo-controlled, randomized, 3-way crossover study is depicted in Supplemental Figure 1. Subjects were randomized to one of the three treatment sequences using a Latin square design. A single dose of daridorexant 25, 50 mg, or placebo was administered at bedtime during the three treatment periods (separated by a washout period of 1 week). In each study period, subjects were discharged from the site in the morning after the nighttime assessments. The protocol was approved by the local ethics committee (Stichting Beoordeling Ethiek Biomedisch Onderzoek, Assen, The Netherlands)—approval number: NL8308.056.22. The study was conducted according to the principles of the Declaration of Helsinki and Good Clinical Practice guidelines, including applicable laws and regulations of The Netherlands.

### PD assessments

**Figure 1.**
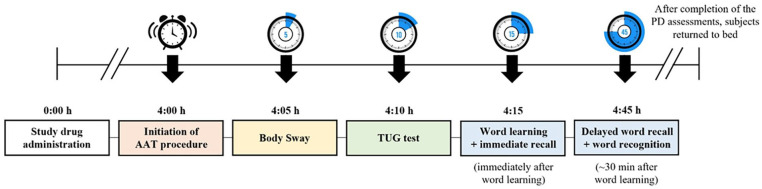
Nighttime PD assessments. Study drugs were administered at least 2 h after the last meal and 30 min before going to bed. AAT: auditory awakening threshold; PD: pharmacodynamic(s); TUG: timed up and go.

Daridorexant effects were assessed on static and dynamic postural stability (body sway and Timed Up and Go (TUG) test), and memory function using the Visual Verbal Learning Test (VVLT). Assessments were performed in the MOTN, 4 h after study drug administration and following forced awakening to determine the auditory awakening threshold (AAT). Immediately after the AAT test, body sway, TUG test, and VVLT were performed. Upon completing the assessments, subjects returned to bed. The flow of the nighttime PD assessments with planned time points (relative to study drug administration) is presented in Figure 1.

The AAT was assessed by introducing a 1000 Hz tone through a calibrated loudspeaker. It commenced at a noise level of 35 dB and increased in 5 dB increments until the subject awakened or the maximum noise level of 100 dB was attained. Each tone was presented for 3 s, with a 15-s silent interval between different noise levels. The outcome measure was the noise level (dB) at which the subjects indicated they were awake. A clinically relevant AAT is indicated at ⩾85 dB, the common noise level emitted by a smoke alarm (Drake et al., 2017).

The main PD endpoint, nocturnal postural stability, was determined by measuring body sway within 5 min after being awakened by the AAT. Subjects stood in a comfortable position with eyes closed using a body sway meter (string attached to the waist), measuring the cumulative extent of anterior–posterior movement (mm) over 2 min (Wright, 1971). At CHDR, the method has been previously used to explore effects of sleep deprivation and following the administration of ethanol, benzodiazepines (Van Steveninck et al., 1993, 1997, 1999), and the orexin receptor antagonists almorexant, daridorexant, and seltorexant (Brooks et al., 2019; Hoever et al., 2010; Muehlan et al., 2018). An increase in body sway of approximately 35% versus placebo observed with alcohol at the legal limit (0.5 g/L) is considered clinically meaningful (Jongen et al., 2014).

The TUG test to assess functional mobility was performed directly after body sway measurements. The subjects raised from a standard armchair, walked for 3 m on a line on the floor, turned around, returned, and sat down again (Podsiadlo and Richardson, 1991). The outcome measure was time (s). A TUG value of ⩾12 s indicates an increased fall risk (Bischoff et al., 2003; Ganz and Lathan, 2020).

The VVLT comprises three distinct components: immediate and delayed word recall, and delayed word recognition. It serves as a method to assess the acquisition and consolidation of information, as well as the active retrieval from long-term memory. The VVLT was performed after the TUG test. Initially, participants were presented with a list of 30 words (commonly used nouns in the subjects’ native language) on a computer screen at a rate of approximately 2 s per word. After the learning phase, participants were tasked to recall as many words as possible (immediate recall). About 30 min later, participants were again asked to recall as many words as they could (delayed recall). Immediately thereafter, participants underwent the delayed word recognition test, in which they were presented with 15 “distractor” words to test memory storage, i.e., the subjects had to discriminate between previously presented words and newly introduced words. The outcome measures of the VVLT included the number of correctly recalled words (for the immediate and delayed recall), and the percentage of correctly recognized words (true positive = previously presented word was correctly identified again; true negative = distractor word correctly identified).

### Safety evaluations

Given the study was performed by administering a single dose of a marketed drug with a well-established safety profile, only a limited number of safety assessments were conducted. This included measurement of vital signs, laboratory assessments including blood chemistry, hematology, and urinalysis, as well as recording of treatment-emergent AEs (TEAEs) and serious AEs (SAEs).

### Statistical analysis

SAS^®^ software, version 9.4 (SAS Institute, Cary, NC, USA) was used for the statistical analysis and the reporting of clinical data. The determination of the sample size was based on a precision estimate, utilizing the mean of paired differences in body sway from a prior daridorexant study conducted in healthy subjects during daytime administration (Berger et al., 2023), as body sway served as the primary outcome variable. It was assumed that with a standard deviation of paired differences in body sway of 269 mm/2 min and an estimated sample size of 24 evaluable subjects, the precision of the 95% confidence interval (CI) for the treatment difference (daridorexant – placebo) would be approximately ±114 mm/2 min (based on the *t*-distribution). Given the 3-way crossover study design and the required balanced distribution of both sex and age groups, the sample size was extended to a total of 36 subjects. Treatment effects on body sway and other PD variables were explored using the treatment difference (daridorexant—placebo) and its 95% CI. Absolute values were analyzed by a linear mixed-effects model including treatment, sequence, period, sex, age group (18−64 and ⩾65 years), and interaction between treatment and age group as fixed effects and subject as random effect. The treatment difference and its 95% CI were calculated from the difference in least squares means (LSMs) and its 95% CI. Missing PD values due to drug effect were imputed based on a worst-case scenario for body sway and TUG test: the highest individual value of the respective treatment, age group, and sex of any subject was assigned for the missing value. For the VVLT, the lowest individual value of the respective treatment, age group, and sex of any subject was assigned for the missing value (correctly recalled/recognized words). For the AAT, a minimum value of 35 dB was assigned to those subjects who were already awake when the AAT procedure started. If a subject did not confirm awakening during any of the presented tones, the maximum 100 dB tone was to be documented as the AAT. Descriptive statistics were determined for baseline demographic characteristics and safety data.

## Results

### Subjects

A total of 36 subjects were included in this study. The mean (range) age of nonelderly adult and elderly subjects was 28.7 (16–60) years and 68.9 (66–72) years, respectively. Overall, the majority (*N* = 34) of the study population was White, the mean (range) body mass index was 24.5 (19.8–33.2) kg/m^2^ and both sex and age groups were equally represented (*N* = 18 nonelderly adults) (*N* = 9 males and *N* = 9 females) and *N* = 18 older adults (*N* = 9 males and *N* = 9 females). All 36 subjects received all study treatments and completed the study, i.e., there were no premature discontinuations. Baseline demographics are summarized in Supplemental Table 1.

### Pharmacodynamics

Overall, there were three missing PD assessments (one body sway, TUG test, and VVLT), all in the same subject due to dizziness related to daridorexant 50 mg, which were imputed as described earlier.

### Auditory awakening threshold

**Figure 2.**
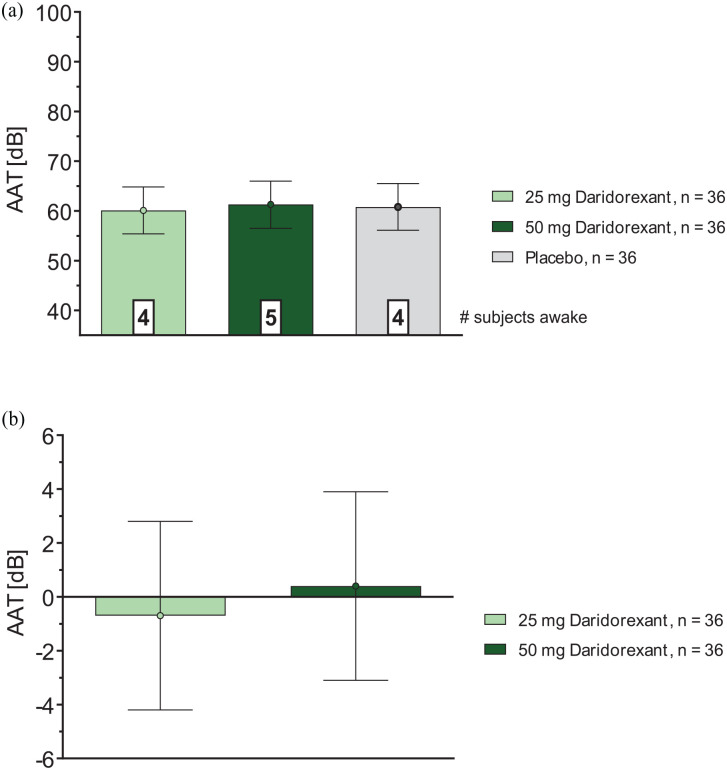
(a) AAT (dB): effects of daridorexant, absolute values, overall population and (b) AAT (dB): effects of daridorexant, differences to placebo, overall population. a) Data are expressed as LSM (95%) CI. b) Data are expressed as ΔLSM (95%). AAT: auditory awakening threshold; CI: confidence interval; dB: decibel; ΔLSM: difference least square means; N: number of subjects.

At 4 h postdose, the average awakening threshold in the overall population was approximately 60 dB (Figure 2(a)) across all three treatments (i.e., 60.1 (55.4, 64.8), 61.3 (56.5, 66.0) and 60.8 (56.1, 65.5) dB for daridorexant 25, 50 mg, and placebo, respectively (Table 1)). Statistical analyses of the absolute values did not detect any difference to placebo (95% CI include zero) for either daridorexant dose (Figure 2(b)), with placebo-corrected LSM (95% CI) of −0.7 (−4.2, 2.8) dB and 0.4 (−3.1, 3.9) dB for daridorexant 25 and 50 mg, respectively (Table 1).

**Table 1.** Summary of the pharmacodynamic assessments, overall population.

Assessment	Unit	25 mg (*N* = 36)	50 mg (*N* = 36)	Placebo (*N* = 36)	25 mg –placebo	50 mg – placebo
		LSM (95%)	LSM (95%)	LSM (95%)	ΔLSM (95%)	ΔLSM (95%)
AAT	(dB)	60.1 (55.4, 64.8)	61.3 (56.5, 66.0)	60.8 (56.1, 65.5)	−0.7 (−4.2, 2.8)	0.4 (-3.1, 3.9)
Body sway	(mm)	332 (264, 400)	361 (293, 429)	295 (227, 364)	36.7 (2.2, 71.3)	65.9 (31.4, 100.4)
TUG test	(s)	6.81 (6.47, 7.16)	7.13 (6.79, 7.48)	6.67 (6.33, 7.02)	0.142 (0.017, 0.267)	0.462 (0.337, 0.587)
VVLT, immediate recall	(correct words)	10.0 (9.0, 11.1)	9.9 (8.8, 11.0)	10.5 (9.5, 11.6)	−0.5 (−0.8, −0.2)	−0.6 (−1.0, −0.3)
VVLT, delayed recall	(correct words)	8.6 (7.1, 10.1)	8.9 (7.4, 10.4)	9.6 (8.1, 11.1)	−1.0 (−1.5, −0.5)	−0.7 (−1.2, −0.2)
VVLT, delayed recognition	(% recognized words)	78.9 (75.1, 82.7)	77.2 (73.4, 81.0)	76.8 (73.0, 80.7)	2.04 (−0.42, 4.49)	0.38 (−2.08, 2.83)

Data are expressed as LSM (95%) and ΔLSM (95%).

AAT: auditory awakening threshold; CI: confidence interval; LSM: least square means; N: number of subjects; TUG test: time up and go test; VVLT: visual verbal learning test.

Similar to the overall population, placebo-corrected AAT values of nonelderly adult and elderly subjects did not differ from placebo for both doses of daridorexant (i.e., LSM (95% CI) of −4.7 (−9.6, 0.2) dB and −3.9 (−8.8, 1.0) dB for nonelderly adult subjects on daridorexant 25 and 50 mg, respectively, and LSM (95% CI) of 3.3 (−1.6, 8.3) dB and 4.7 (−0.2, 9.6) dB for elderly subjects on daridorexant 25 and 50 mg, respectively (Supplemental Table 2)).

The number of subjects already awake prior to initiation of the first tone at the lowest noise level of 35 dB was balanced across age groups and treatments, i.e., *N* = 4 for daridorexant 25 mg, *N* = 5 for daridorexant 50 mg, and *N* = 4 for placebo (Figure 2(a)). No subject remained unresponsive to the maximum sound level of 100 dB. Only one subject (on daridorexant 50 mg) required the maximum tone of 100 dB to be played once before waking up, and no subject needed to be forcibly awakened by a study technician.

### Body sway

**Figure 3.**
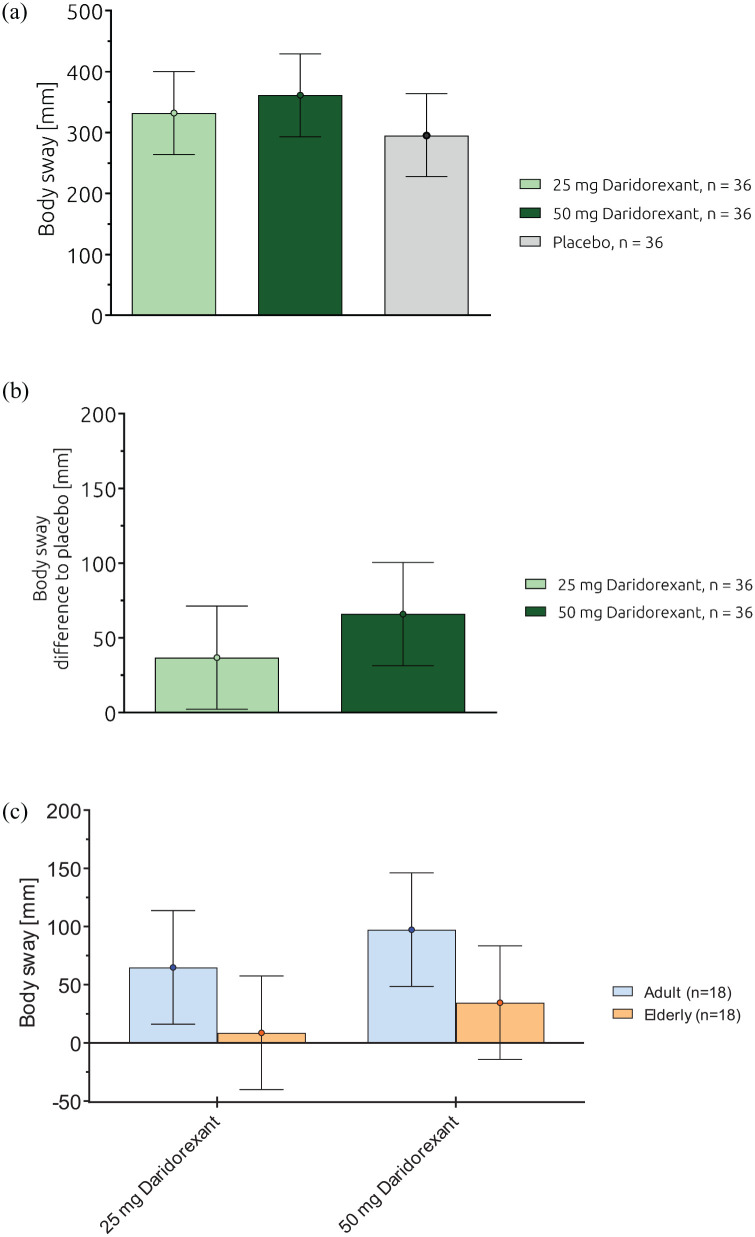
(a) Body sway (mm): effects of daridorexant, absolute values, overall population, (b) Body Sway (mm): effects of daridorexant, differences to placebo, overall population, and (c) Body Sway (mm): effects of daridorexant, differences to placebo, by age group. a) Data are expressed as LSM (95%) CI. b) and c) Data are expressed as ΔLSM (95%). CI: confidence interval; ΔLSM: difference least square means; mm: millimeter; N: number of subjects.

Absolute values for body sway in LSM (95% CI) were 332 (264, 400) mm, 361 (293, 429) mm, and 295 (227, 364) mm for daridorexant 25, 50 mg, and placebo, respectively (Figure 3(a), Table 1). In comparison to placebo, body sway in the overall population increased dose-dependently with differences in LSM (95% CI) of 36.7 (2, 71) mm and 65.9 (31, 100) mm for daridorexant 25 and 50 mg, respectively (Figure 3(b), Table 1).

In nonelderly adults as well as in elderly subjects, mean body sway values were more pronounced for daridorexant compared to placebo, showing a weak trend for dose-dependency (Figure 3(c)). Placebo-corrected body sway values (LSM, 95% CI) in nonelderly adult subjects showed differences to placebo of 64.8 (16.0, 113.7) mm and 97.3 (48.4, 146.1) mm for daridorexant 25 and 50 mg, respectively, while elderly subjects showed no differences (95% CIs include zero) to placebo with LSM (95% CI) of 8.6 (−40.2, 57.5) and 34.6 (−14.3, 83.4) mm. (Figure 3(c), Supplemental Table 2).

### Timed up and go

In the overall population, subjects completed the test in approximately 6–8 s across all three treatments (i.e., LSM (95% CI) of 6.81 (6.47, 7.16), 7.13 (6.79, 7.48), and 6.67 (6.33, 7.02) s for daridorexant, 25, 50 mg, and placebo, respectively (Table 1)). Compared to placebo, the results indicated a small dose-dependent increase with a difference in LSM (95% CI) of 0.142 (0.017, 0.267) s and 0.462 (0.337, 0.587) s for daridorexant 25 and 50 mg, respectively (Supplemental Figure 2, Table 1).

Based on absolute values in the placebo condition, nonelderly adults generally completed the TUG test approximately 1–2 s faster than elderly subjects, with no meaningful differences to placebo (<1 s) in both age groups (Supplemental Table 2).

### Visual verbal learning test

In the overall population, subjects correctly recalled approximately 10 words immediately after word learning, (i.e., 10.0 (9.0, 11.1), 9.9 (8.8, 11.0) and 10.5 (9.5, 11.6) correct words for daridorexant 25, 50 mg, and placebo, respectively (Table 1)). Statistical analyses of the absolute values revealed a decrease of correctly recalled words compared to placebo with a difference in LSM (95% CI) of −0.5 (−0.8, −0.2) and −0.6 (−1.0, −0.3) words for daridorexant 25 and 50 mg, respectively (Supplemental Figure 3(a), Table 1).

For delayed recall, i.e., the second component of the VVLT, absolute values of correctly recalled words were approximately 9 words after a period of 30 min across treatments (i.e., 8.6 (7.1, 10.1), 8.9 (7.4, 10.4) and 9.6 (8.1, 11.1) correct words for daridorexant 25, 50 mg, and placebo, respectively (Table 1)). Similar to the immediate recall, differences to placebo for delayed recall were small and not dose-dependent, with a decrease in LSM (95% CI) of correctly recalled words versus placebo of −1.0 (−1.5, −0.5) and −0.7 (−1.2, −0.2) words for daridorexant 25 and 50 mg, respectively (Supplemental Figure 3(b), Table 1).

Differences were observed in both immediate and delayed recall when comparing absolute values of nonelderly adult and elderly subjects, i.e., nonelderly adults recalled more words (2–3 words during immediate recall and 4–5 words during delayed recall) compared to elderly subjects with either treatment, including placebo (Supplemental Table 2). Differences to placebo were small for both age groups and did not yield a trend for any influence of age on daridorexant-related effects on word recall (Supplemental Table 2).

For delayed word recognition, i.e., the third component of the VVLT, the percentages of correctly recognized words (i.e., true positive and true negative) were similar across all treatments (LSM (95% CI) 78.9 (75.1, 82.7), 77.2 (73.4, 81.0), and 76.8 (73.0, 80.7) % for daridorexant 25, 50 mg, and placebo, respectively (Table 1)), with no difference to placebo for either daridorexant dose (Supplemental Figure 3(c), Table 1).

Similar to word recall, nonelderly adults were able to correctly recognize more words (absolute values) compared to elderly subjects with no meaningful differences between daridorexant and placebo for either age group (Supplemental Table 2).

### Safety

Overall, TEAEs were distributed across treatments (*N* = 13 with daridorexant 25 mg, *N* = 17 with daridorexant 50 mg, and *N* = 11 with placebo), affecting a total of 17 subjects (42%) and were reported in a similar proportion in nonelderly adults and elderly subjects. Table 2 provides details on all TEAEs reported by two or more subjects during the study. The most frequently reported AEs included headache, nausea, and dizziness, which were also reported following placebo treatment, while somnolence and dry mouth were only reported following treatment with daridorexant. All AEs were of mild or moderate intensity, with no severe or SAEs reported, and all resolved without sequelae, and none led to study discontinuation. Furthermore, vital signs or laboratory variables were within normal or acceptable ranges, following administration of daridorexant and placebo.

**Table 2.** Summary of treatment-emergent adverse events reported in two or more subjects during the study.

Preferred term	25 mg (*N* = 36)	50 mg (*N* = 36)	Placebo (*N* = 36)
	*N* (%)	*N* (%)	*N* (%)
Headache	3 (8.3)	4 (11.1)	3 (8.3)
Nausea	2 (5.6)	2 (5.6)	5 (13.9)
Dizziness	1 (2.8)	2 (5.6)	1 (2.8)
Somnolence	1 (2.8)	3 (8.3)	0
Dry mouth	0	2 (5.6)	0

Data are expressed as *N* (%).

*N*: number of subjects.

## Discussion

Results from this study indicate that daridorexant administration in the evening preserves the ability to be awakened by external noise stimuli and does not affect postural stability, functional mobility, and cognitive function of healthy subjects, including elderly, to a clinically meaningful extent in the MOTN.

When addressing the need for pharmacological treatment of insomnia, the challenge is to facilitate sleep without compromising awakening, while also maintaining the patient’s functional mobility (both standing and walking) and cognitive functions during nighttime awakenings and the following morning. For the latter, no next-morning residual effects have been shown with daridorexant in insomnia patients in larger studies (Dauvilliers et al., 2020; Kunz et al., 2023; Mignot et al., 2022; Zammit et al., 2020). Rather improved daytime functioning versus placebo was observed, specifically for daridorexant 50 mg (Luyet et al., 2023; Mignot et al., 2022). Considering the well-established age-related variations in sleep patterns (Ohayon et al., 2004; Zepelin et al., 1984), spontaneous awakenings and prolonged wake periods during the night are more prevalent in middle-aged and elderly people (Zepelin et al., 1984). These individuals often need to get up during the night due to, e.g., nocturia, and thus, making the elderly population particularly susceptible to falls during nighttime awakenings (Hill et al., 2007; Mendelson, 1996; Tinetti et al., 1988; Treves et al., 2018). Falls in the elderly are often linked to reduced muscular strength or cognitive function, which can be exacerbated by CNS-depressant drugs. Based on these safety concerns, physicians often may refrain from prescribing sleep medicines (Bischoff et al., 2003; Ganz and Lathan, 2020). The American Geriatrics Society Beers Criteria explicitly recommend avoiding benzodiazepines and Z-drugs in older adults due to potential AEs such as falls and fractures (American Geriatrics Society Beers Criteria^®^, 2023). Therefore, there is a need for pharmacological insomnia treatments that do not cause a clinically meaningful decrease in functional mobility or cognition.

In previous safety studies conducted in healthy subjects in the MOTN, widely used sleep-promoting drugs have been found to increase the external sound threshold required to awaken (Drake et al., 2017). Following bedtime administration of zolpidem 10 mg, over 60% of subjects showed no response to noise stimuli of up to 110 dB (a noise level at a live rock concert), with 30% of subjects necessitating forced manual awakening by a study technician (Drake et al., 2017). In contrast, following bedtime administration of daridorexant 25 and 50 mg in the present study, all subjects regardless of age, awakened independently at an average noise level of approximately 60 dB (level of a normal office conversation) following the external auditory stimulus, with no subject requiring to be awakened by a study technician. Notably, the mean AAT recorded for daridorexant was much lower than the mean AAT of 103 dB previously reported with zolpidem 10 mg, and, even more importantly, substantially lower than the volume of 85 dB emitted by standard smoke detectors (Drake et al., 2017). These findings are reassuring and consistent with the results observed with other DORAs such as suvorexant and lemborexant, demonstrating placebo-like effects in similar studies assessing the AAT (Drake et al., 2017; Murphy et al., 2020). Overall, these findings affirm the safety profile of DORAs in terms of preservation of sleep arousability (differently from zolpidem’s impairment), and are consistent with data from animal models showing that sleep promoted by daridorexant can be immediately surmounted (Roch et al., 2021). At the time of the AAT assessment, 4, 4, and 5 subjects were already awakened when presenting the lowest noise level following treatment with placebo, daridorexant 25 mg, and daridorexant 50 mg, respectively. This finding is not unexpected, as the study was conducted in healthy subjects whose sleep cannot not be further improved. This also indirectly demonstrates the mechanism of action of daridorexant, which only exhibits its effects on sleep when the orexin system is disturbed. This aligns with the hypothesis proposed by Drake et al. (2017) suggesting that CNS-depressant medications such as zolpidem, triazolam, and flurazepam increase arousal thresholds, whereas wake-inhibiting medications such as DORAs do not.

The observed differences in body sway in the present study of approximately 12% and 22% between daridorexant 25 and 50 mg, respectively, and placebo, are deemed to have no meaningful impact on common nighttime activities. The absolute values of observed body sway were in line with findings from prior studies in which daridorexant was administered during the day (Berger et al., 2020; Muehlan et al., 2018, 2019). Previous clinical studies involving widely used sleep-promoting sedatives (e.g., zolpidem, zopiclone) led to a significant and clinically meaningful impairment of postural stability in the MOTN (Murphy et al., 2020, Vermeeren et al., 2015). Postural instability has been shown to be associated with an elevated risk of falling (Fernie et al., 1982). Johansson et al. (2017) reported that an increase in body sway predicted future incident falls in 1877 community-dwelling elderly females and males. The researchers showed that fall risk approximately doubled with an odds ratio (95% CI) of 1.90 (1.12–3.22) during a 1-min eyes-closed measurement. It is important to highlight that Johansson et al. (2017) used a slightly different assay (Wii Balance Board, Nintendo) compared to the ataxiameter used in studies with lemborexant and also in the present study with daridorexant. Although indirectly comparing PD results across studies using different assessment methods is challenging and should be done with caution, it is worth noting that nocturnal body sway increased in a clinically meaningful manner by 47% and >100% versus placebo with lemborexant 10 mg and zolpidem 6.5 mg, respectively (Murphy et al., 2020). Nocturnal body sway with zolpidem 10 mg has been shown to increase by up to 380% (Otmani et al., 2012).

In the present study, the increase in body sway observed in the overall population following daridorexant administration appeared to be driven by nonelderly adult subjects, while effects in the elderly population were similar to those of placebo. From previous studies, it is known that daridorexant exposure is approximately 35% higher in the elderly population compared to young adults (Muehlan et al., 2020b). Nonetheless, studies involving the elderly (both healthy elderly and elderly insomnia patients) have demonstrated no correlation between elevated daridorexant plasma concentrations in the elderly and a change from baseline in safety endpoints evaluated in the next morning (Fietze et al., 2022; Muehlan et al., 2020a, 2020b, Zammit et al., 2020).

The absence of meaningful residual next-day PD effects in the elderly despite a higher exposure compared to young adults affirms there is no necessity to adjust the dose in the elderly population (Fietze et al., 2022). This may provide a safeguard for treating physicians when prescribing daridorexant, especially to the elderly patient population. In addition, the absence of clinically meaningful effects on nighttime postural stability in elderly is particularly important, given the consistently higher body sway and related fall risk associated with commonly prescribed sleep-promoting drugs, e.g., benzodiazepines or zolpidem (Drake et al., 2017; Ganz and Lathan, 2020; Mendelson, 1996; Murphy et al., 2020; Seppala et al., 2018; Zammit et al., 2009).

When daridorexant effects on functional mobility were assessed, the average completion time for the TUG test was 6–8 s across all 3 treatments, remaining well below the established cut-off value of 12 s which indicates below-normal performance (Bischoff et al., 2003; Ganz and Lathan, 2020). Treatment differences (daridorexant-placebo) in both nonelderly adult and elderly subjects were small (⩽1 s) and not clinically relevant, based on the 12 s threshold indicating a higher fall risk. There were no instances of falls in either age group across all three treatment periods, indicating safe conduct of the nocturnal balance and mobility assessments.

With regard to the evaluation of nocturnal cognitive function (memory), clinically meaningful effects have been reported for both zolpidem and lemborexant (Dingemanse et al., 1995; Murphy et al., 2020). These effects are consistent with the prescribing information of the two drugs (AMBIEN CR^®^ USPI, 2016; DAYVIGO™, 2019), and contrast with the findings observed in this study, such that daridorexant showed only small differences versus placebo of up to 1 correctly recalled word (approximately 10%), all considered to be of no clinical relevance.

Overall, the findings of this phase 1 study in healthy nonelderly adults and elderly subjects contribute to the extensive body of PD data generated with daridorexant. They demonstrate that both daridorexant 25 and 50 mg pose no clinically meaningful deteriorations in nocturnal postural stability, functional mobility (both standing and walking), and cognitive function/memory after forced awakening in the MOTN. The administration of daridorexant 25 and 50 mg coupled with forced awakening and nighttime PD assessment was safe, well-tolerated, and the reported AEs were in line with the established safety profile in healthy subjects. The crossover study design and the chosen population of healthy subjects reduced intersubject variability across PD assessments and increased precision of measurements, thereby ensuring more robust data. Furthermore, a balanced 1:1 age ratio allowed for conclusions related to age. Limitations of this PD study include the absence of a positive control to confirm assay sensitivity and the absence of sleep EEG data to provide insights into the sleep stage of the subjects at the time of forced awakening as well as the return-to-sleep latency after the nighttime assessments. In addition, it is not known whether these results in healthy noninsomnia subjects would be similar in the relevant patient population suffering from insomnia.

## Conclusions

Following bedtime administration, daridorexant preserved the awakening from sleep, which is crucial for emergencies (such as fires) during the night. Furthermore, daridorexant did not compromise postural stability, functional mobility, or cognitive function to a clinically relevant extent in the MOTN. When translating the results of this study in healthy subjects to patients with insomnia, caution is warranted but negative effects of daridorexant on safety after nighttime awakenings are considered unlikely, also in an insomniac patient population.

## Supplemental Material

sj-docx-1-jop-10.1177_02698811241293997 – Supplemental material for Nighttime safety of daridorexant: Evaluation of responsiveness to an external noise stimulus, postural stability, walking, and cognitive function Supplemental material, sj-docx-1-jop-10.1177_02698811241293997 for Nighttime safety of daridorexant: Evaluation of responsiveness to an external noise stimulus, postural stability, walking, and cognitive function by Massimo Magliocca, Ingrid Koopmans, Cedric Vaillant, Vincent Lemoine, Rob Zuiker, Jasper Dingemanse and Clemens Muehlan in Journal of Psychopharmacology
