# Supplementary material for: Nighttime safety of daridorexant: Evaluation of responsiveness to an external noise stimulus, postural stability, walking, and cognitive function
Source: J Psychopharmacol. 2024 Dec 6;39(3):223–32. doi: 10.1177/02698811241293997 (PMC11843790; doi:10.1177/02698811241293997)
Supplement: sj-docx-1-jop-10.1177_02698811241293997 – Supplemental material for Nighttime safety of daridorexant: Evaluation of responsiveness to an external noise stimulus, postural stability, walking, and cognitive function [file sj-docx-1-jop-10.1177_02698811241293997.docx]

**Supplemental Material**

**Supplemental Figure 1 Study Design**


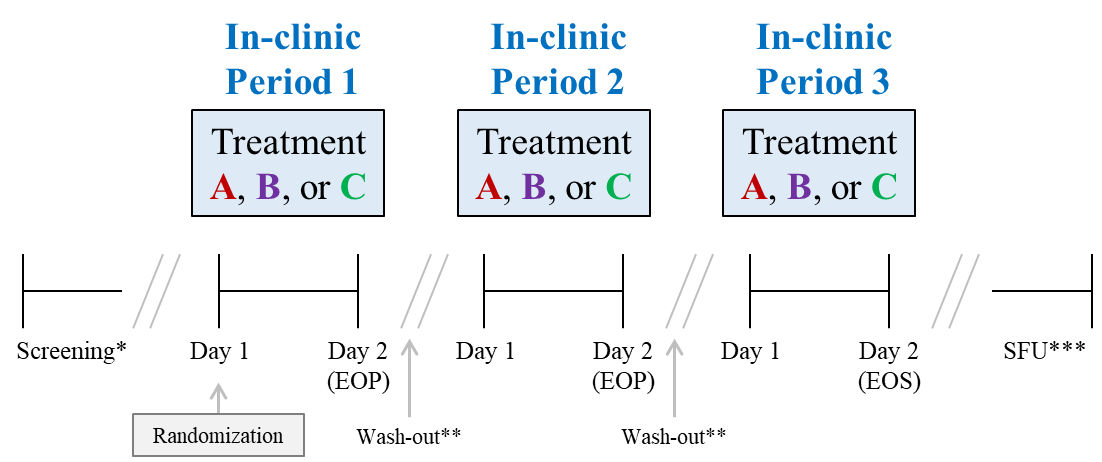


Treatment A, B, and C reflect 25 mg daridorexant, 50 mg daridorexant, and placebo, respectively. *Screening evaluation was performed within 21 to 3 days for men and women of non-childbearing potential or within 28 to 10 days for women of childbearing potential. **The washout period between EOP of the preceding period and Day 1 of the following period was at least 1 week. ***A SFU telephone call took place 5–7 days after EOS. EOP = End-of-Period; EOS = End-of-Study; SFU = Safety Follow-Up.

**Supplemental Figure 2 TUG Test [s]: effects of daridorexant, differences to placebo, overall population**

Data are expressed as ΔLSM (95% CI). TUG = Timed Up and Go; CI = confidence interval; sec = seconds; ΔLSM = difference least square means; N = number of subjects.

**Supplemental Figure 3A VVLT, immediate recall [correct words]: effects of daridorexant, differences to placebo, overall population**

Data are expressed as ΔLSM (95% CI). VVLT = Visual Verbal Learning Test; CI = confidence interval; ΔLSM = difference least square means; N = number of subjects.

**Supplemental Figure 3B VVLT, delayed recall [correct words]: effects of daridorexant, differences to placebo, overall population**

Data are expressed as ΔLSM (95% CI). VVLT = Visual Verbal Learning Test; CI = confidence interval; ΔLSM = difference least square means; N = number of subjects.

**Supplemental Figure 3C VVLT, delayed recognition [% correctly recognised words]: effects of daridorexant, differences to placebo, overall population**

Data are expressed as ΔLSM (95% CI). VVLT = Visual Verbal Learning Test; CI = confidence interval; ΔLSM = difference least square means; N = number of subjects.

**Supplemental Table 1 Baseline demographic characteristics**

| Characteristic |  | All-treated set [N = 36] |
| --- | --- | --- |
|  |  |  |
|  |  |  |
| **Sex** | **[N(%)]** |  |
| Female |  | 18 (50.0) |
| Male |  | 18 (50.0) |
|  |  |  |
| **Age group** | **[N(%)]** |  |
| Non-elderly adult  (< 65 years) |  | 18 (50.0) |
| Elderly  (≤ 65 years) |  | 18 (50.0) |
|  |  |  |
| **Age, years** | **[mean (SD)]** | 48.8 (22.2) |
| Non-elderly adult  (< 65 years) | **[mean (SD)]** | 28.7 (12.3) |
| Elderly  (≤ 65 years) | **[mean (SD)]** | 69.9 (2.31) |
|  |  |  |
| **Weight, kg** | **[mean (SD)]** | 74.5 (11.8) |
| **BMI, kg/m2** | **[mean (SD)]** | 24.5 (3.08) |
|  |  |  |

Arithmetic mean is presented. BMI = body mass index; N = number of subjects;
SD = standard deviations.

**Supplemental Table 2 Summary of the pharmacodynamic assessments, by age groups**

| **Assessment** | **Unit** | **Population** | **25 mg [N = 18]** | **50 mg [N = 18]** | **Placebo [N = 18]** | **25 mg - Placebo** | **50 mg - Placebo** |
| --- | --- | --- | --- | --- | --- | --- | --- |
|  |  |  | LSM (95%) | LSM (95%) | LSM (95%) | ΔLSM  (95%) | ΔLSM  (95%) |
|  |  |  |  |  |  |  |  |
| **AAT** | **[dB]** | **Non-elderly adults** | 57.8  (51.1, 64.4) | 58.6  (52.0, 65.3) | 62.5  (55.8, 69.2) | –4.7 (–9.6, 0.2) | –3.9 (–8.8, 1.0) |
|  |  | **Elderly** | 62.5 (55.8, 69.2) | 63.9 (57.2, 70.5) | 59.2 (52.5, 65.8) | 3.3  (–1.6, 8.3) | 4.7 (–0.2, 9.6) |
| **Body Sway** | **[mm]** | **Non-elderly adults** | 343 (247, 439) | 375 (279, 472) | 278 (182, 375) | 64.8 (16.0, 113.7) | 97.3 (48.4, 146.1) |
|  |  | **Elderly** | 321 (225, 418) | 347 (251, 444) | 313 (216, 409) | 8.64 (–40.18, 57.46) | 34.6 (–14.3, 83.4) |
| **TUG Test** | **[s]** | **Non-elderly adults** | 5.93 (5.44, 6.42) | 6.20 (5.71, 6.68) | 5.90 (5.41, 6.38) | 0.033 (-0.144, 0.209) | 0.298 (0.122, 0.475) |
|  |  | **Elderly** | 7.70 (7.21, 8.18) | 8.07 (7.58, 8.56) | 7.45 (6.96, 7.93) | 0.251 (0.074, 0.428) | 0.625 (0.448, 0.802) |
| **VVLT,  immediate recall** | **[correct words]** | **Non-elderly adults** | 11.4 (9.9, 13.0) | 12.2 (10.7, 13.8) | 12.3 (10.8, 13.8) | –0.9 (–1.3, –0.4) | –0.1 (–0.6, 0.4) |
|  |  | **Elderly** | 8.6 (7.1, 10.1) | 7.9 (6.4, 9.4) | 8.7 (7.2, 10.2) | –0.1 (–0.6, 0.3) | –0.8 (–1.3, –0.4) |
| **VVLT,  delayed recall** | **[correct words]** | **Non-elderly adults** | 11.3 (9.3, 13.4) | 12.3 (10.2, 14.4) | 12.6 (10.5, 14.7) | –1.3 (–2.0, –0.6) | –0.3 (–1.0, 0.4) |
|  |  | **Elderly** | 5.8 (3.8, 7.9) | 5.9 (3.8, 8.0) | 6.6  4.5, 8.7) | –0.8 (–1.5, –0.1) | –0.7 (–1.4, 0.0) |
| **VVLT,  delayed recognition** | **[%recognised words]** | **Non-elderly adults** | 83.52 (78.12, 88.91) | 82.96 (77.56, 88.35) | 81.29 (75.89, 86.69) | 2.23 (–1.25, 5.70) | 1.67 (–1.81, 5.14) |
|  |  | **Elderly** | 74.24 (68.85, 79.64) | 71.48 (66.09, 76.88) | 72.40 (67.00, 77.80) | 1.84 (–1.63, 5.32) | –0.92 (–4.39, 2.56) |
|  |  |  |  |  |  |  |  |

Data are expressed as LSM (95%) and ΔLSM (95%). AAT = auditory awakening threshold; CI = confidence interval; LSM = least square means; N = number of subjects; TUG test = Time Up and Go Test; VVLT = Visual Verbal Learning Test.
